# First record of *Rhoptrocentrus piceus* Marshall (Hymenoptera, Braconidae, Doryctinae) as parasitoid of *Psacothea hilaris hilaris* (Pascoe) (Coleoptera, Cerambycidae)

**DOI:** 10.3897/zookeys.482.8946

**Published:** 2015-02-10

**Authors:** Augusto Loni, Costanza Jucker, Sergey Belokobylskij, Daniela Lupi

**Affiliations:** 1 Department of Agriculture, Food and Environment, Pisa University, Via del Borghetto, 80, 56124 Pisa, Italy; 2 Department of Food, Environmental and Nutritional Sciences, University of Milan, Via Celoria 2, 20133 Milano, Italy; 3 Zoological Institute Russian Academy of Sciences, St. Petersburg, 199034, Russia; Museum and Institute of Zoology, Polish Academy of Sciences, Wilcza 64, Warszawa 00–679, Poland; 4 Museum and Institute of Zoology, Polish Academy of Sciences, Wilcza 64, Warszawa 00–679, Poland

**Keywords:** Ectoparasitoid, new record, biocontrol, exotic pest

## Abstract

The species *Rhoptrocentrus piceus* Marshall (Hymenoptera: Braconidae) was reared from the larvae of the xylophagous beetle *Psacothea hilaris hilaris* (Pascoe) (Coleoptera: Cerambycidae), an exotic pest of *Ficus* and *Morus* species native to eastern Asia. It was recorded in the north of Italy in September 2005. This discovery is the first report of this species as parasitoids of the yellow spotted longicorn beetle all over the world.

## Introduction

The invasion of new exotic species is an increasing phenomenon in all European countries. One of the main reason is the great increase of import and export goods, including living plant material, throughout the world. Italy is particularly vulnerable due to the structure of its territory. It extends over a wide latitude, with numerous mountain ranges along its length. Such territory structure creates a great variability in micro-climatic conditions. The presence of many different ecosystems in such a rich geo-morphological and climatic context can facilitate the settlement of new invasive species (Frasconi et al. 2013). It has been estimated that approximately 200 exotic species have settled in Italy since 1970 and the highest number of new records, 111 new species, was registered in the decade 1991-2000 (Longo 2009, Jucker and Lupi 2011).

The initial success of an exotic pest is due to the interaction of the biological performance of the species with habitat characteristics (Grobler and Lewis 2008, Jucker and Lupi 2011). When an exotic pest colonizes a new habitat, native potential natural parasites need time to find, recognize, and adapt to the new host species.

Among the pests recently detected in Italy, there is the yellow spotted longicorn beetle *Psacothea hilaris hilaris* (Pascoe) (Coleoptera: Cerambycidae: Lamiinae: Lamiini), an exotic pest of *Morus* and *Ficus* trees. The insect is native to eastern Asia (Kim et al. 2009) and was detected in Europe for the first time in Italy in 2005, where it has now become established (Jucker et al. 2006, Lupi et al. 2013). In 2012 the beetle was also recorded in Germany (EPPO 2012). In the native countries the insect is mostly associated with mulberry trees, whereas in Italy it prefers fig trees (Lupi et al. 2013). The pest larvae tunnel into the xylem of host trees after a first period of feeding under the bark. This results in considerable damage to the tree, which is progressively weakened until death. The adults feed on the leaves and on the tender bark of the smaller branches. Studies of the biology in the native countries indicated that *Psacothea hilaris hilaris* is generally univoltine, but depending on the time of oviposition, it could be also bivoltine (Watari et al. 2002).

As studies on natural enemies are few also in its native countries (Hong et al. 2008), a long-term study has been carried out to improve the knowledge on *Psacothea hilaris hilaris* relationships with autochthonous natural enemies in Italy. The present paper reports the results of a survey that was carried out in an area where *Psacothea hilaris hilaris* is present since 2006.

## Materials and methods

In order to acquire data on the presence of autochthonous natural enemies, surveys were carried out on plants infested by *Psacothea hilaris hilaris* in summer 2013, at two sites in the locality of Erba (Como municipality, Italy) [45°49'40.06"N, 9°13'07.44"E; 45°48'06.78"N; 9°13'02"E].

A visual analysis of the infested fig trees was first performed. The observation of sawdust was the evidence of the presence of *Psacothea hilaris hilaris* preimaginal instars. Branches were cut from infested plants in different sites, transferred to the laboratory, and stored inside cages at room temperature. Some larvae were removed and checked whit the stereomicroscope to confirm *Psacothea hilaris hilaris* presence using the key proposed by Pennacchio et al. (2012). Cages were controlled weekly to check the presence of parasitoids and the emergence of *Psacothea hilaris hilaris* or other bark beetles from the logs.

The emerged specimens of Hymenoptera were collected, stored as dry material as well as in alcohol (70%) and classified to species level following Marsh (1997) and Belokobylskii (2001).

## Results and discussion

From the logs collected in one locality in Erba (Como municipalities) [45°48'06.78"N; 9°13'02"E] on 10 September 2013, 29 females (no males) emerged of *Rhoptrocentrus piceus* Marshall (Hymenoptera: Braconidae: Doryctinae): four specimens in November 2013 and the others in April 2014. In the same logs only adults of *Psacothea hilaris hilaris* were registered.

**Figure 1.**
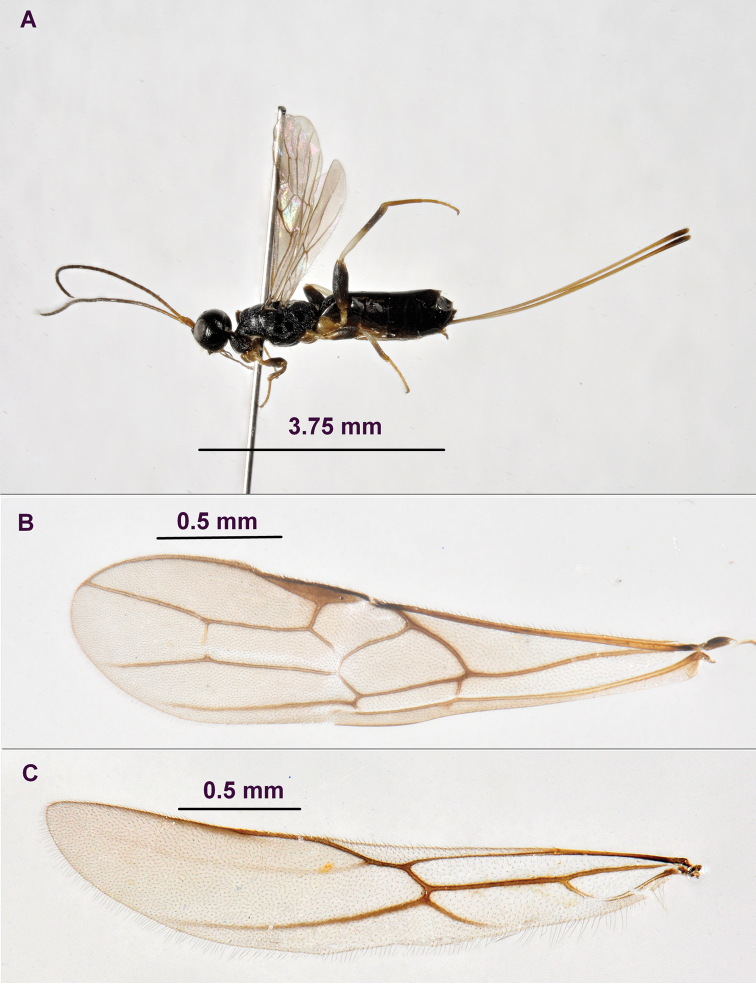
*Rhoptrocentrus piceus* Marshall: **A** habitus, lateral view **B** fore wing **C** hind wing.

**Figure 2.**
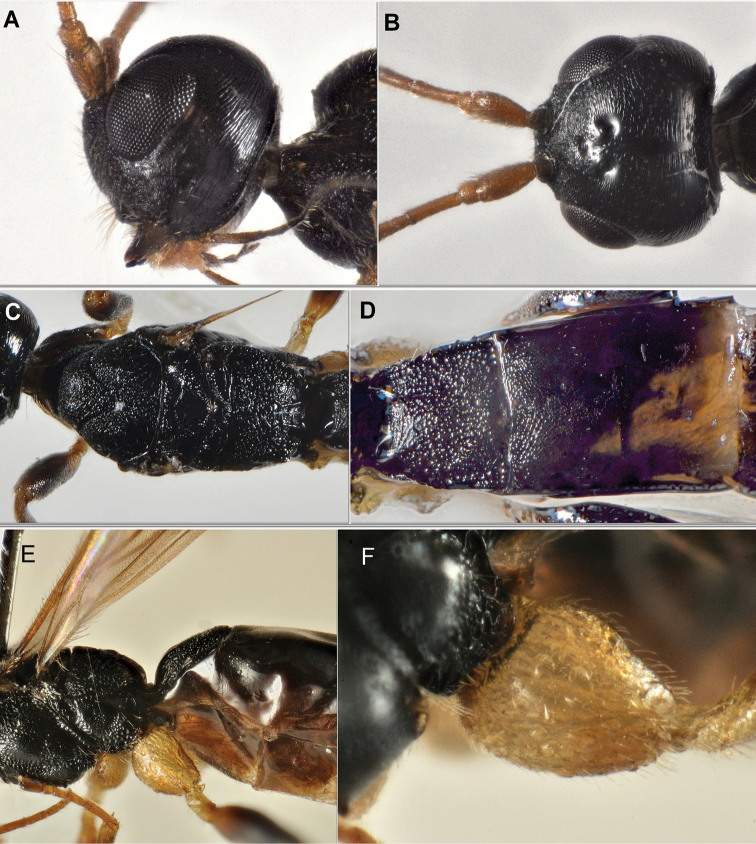
*Rhoptrocentrus piceus* Marshall: **A** head, lateral view **B** head, dorsal view **C** mesosoma, dorsal view **D** metasoma, first three segments, dorsal view **E** propodeum and base of metasoma, lateral view **F** hind coxa, lateral view.

The body length of *Rhoptrocentrus piceus* ranged from 2.75 mm to 4.86 mm, confirming the high variability of the body size for this species (Becker 1979, Belokobylskij 2001) (Table 1). The main features of the genus and the species were confirmed by consulting the most recent keys (Marsh 1997, Belokobylskij 2001, Belokobylskij and Maeto 2009). Terminology adopted for morphological features and measurements follows Belokobylskij and Maeto (2009) (Figs 1 and 2).

**Table 1.** Body size variability of the *Rhoptrocentrus piceus* specimens emerged from larvae of *Psacothea hilaris hilaris*.

	Body length (mm) (N = 29)	Ovipositor length (mm) (N = 23)
Mean ± SD	3.7 ± 0.63	3.15± 0.62
Maximum value	4.865	4.49
Minimum value	2.75	2.04

The genus *Rhoptrocentrus* belongs to the tribe Doryctini including approximately 35 Palaearctic genera (Belokobylskij et al. 2004). This is a moderately large subfamily of the family Braconidae with more than 1000 described species worldwide. Most of the known doryctine species are idiobiont gregarious ectoparasitoids of the larvae of xylophagous or bark-boring Coleoptera, while some species live on Lepidoptera or Hymenoptera-Symphyta (sawfly) larvae. Exceptionally they were reared from adults of Embiopter or living within termites nests and several Neotropical genera behave as phytophagous or gall-associated wasps (Marsh 1997, Belokobylskij et al. 2004, Zaldivar-Riveron et al. 2014).

The genus *Rhoptrocentrus* includes only three described species, *Rhoptrocentrus piceus* Marshall with a Holarctic distribution (Yu et al. 2012), *Rhoptrocentrus cleopatrae* Belokobylskij, so far known only from Egypt (Belokobylskij 2001), and *Rhoptrocentrus yarramanensis* Belokobylskij, Iqbal et Austin, recently described from Australia (Belokobylskij et al. 2004). *Rhoptrocentrus piceus* is relatively common in the western Palaearctic, but in its eastern part this species was recorded only from Japan (when it was secondarily described under the name *Doryctomorpha chlorophori*: Watanabe 1951), with large gaps of its distribution in the eastern part of Russia between the Urals and Japan. Interestingly, *Rhoptrocentrus piceus* again appeared in north Vietnam (first record: 1 female, ”Vietnam: Hoa Binh Province, Yen Thuy District, Da Phuc, 20°18'N 105°35'E, h=100 m, 3–4.05.2002, S. Belokobylskij”; 1 female, ”Vietnam: Vinh Phuc Prov., Me Linh District, Ngoc Thanh, Tam Dao foothill, 21°24'N 105°43'E, h=400 m, 12-13.05.2002, S. Belokobylskij”; both specimens from Zoological Institute, St Petersburg, Russia). This species was also discovered in the Nearctic region (several states of the USA). The genus *Rhoptrocentrus* was already referred to from Mexico (Coronado-Blanco 2013), but without species names; here we record *Rhoptrocentrus piceus* from Mexico for the first time: 1 female, “Mexico. Tamaulipas, Altamira, Ej. Aquiles Serdan, Trampa Malaise 3, 22°33'2.78"N, 97°54'13.11"O, 15–30 Marzo, 2013”; 1 female, “Monterrey, Nuevo Leon, 20-IV-86, E. Ruiz C.” (both specimens from the collection of the Universidad Autonoma de Tamaulipas, Cd. Victoria, Mexico).

This species has a wide range of hosts mainly belong to the orders of Coleoptera [families Anobiidae, Bostrichidae, Buprestidae, Cerambycidae, and Curculionidae (including Scolytinae)], but also to Hymenoptera (Xiphydriidae) and Lepidoptera (Coleophoridae and Tortricidae) (Belokobylskij and Maeto 2009, Belokobylskij and Zikic 2009, Yu et al. 2012, Zikic et al. 2013). The host range of this parasitoid, as well as its wide distribution across all the Holarctic region, with penetration into the Oriental region, suggest a high ability in adapting to different ecological conditions. All these characteristics make it a very suitable parasitoid for the containment of new wood-boring invasive species representing an increasing problem across the Italian territories (Loni et al. 2012). Our finding of this wasp on the new exotic pest *Psacothea hilaris hilaris* seems to validate such a consideration and encourages further studies regarding the biology of *Rhoptrocentrus piceus* as well as the possibility to mass rear it (Turgeons and Smith 2013).
